# Evaluating Manifest Monotonicity Using Bayes Factors

**DOI:** 10.1007/s11336-015-9475-8

**Published:** 2015-09-16

**Authors:** Jesper Tijmstra, Herbert Hoijtink, Klaas Sijtsma

**Affiliations:** Department of Methodology and Statistics, Faculty of Social and Behavioral Sciences, Tilburg University, PO Box 90153, 5000 LE Tilburg, The Netherlands; CITO, NATIONAL Institute for Educational Measurement, Arnhem, The Netherlands; Utrecht University CITO, NATIONAL Institute for Educational Measurement, Utrecht, The Netherlands; Tilburg University, Tilburg, The Netherlands

**Keywords:** Bayes factor, essential monotonicity, item response theory, latent monotonicity, manifest monotonicity

## Abstract

The assumption of latent monotonicity in item response theory models for dichotomous data cannot be evaluated directly, but observable consequences such as manifest monotonicity facilitate the assessment of latent monotonicity in real data. Standard methods for evaluating manifest monotonicity typically produce a test statistic that is geared toward falsification, which can only provide indirect support in favor of manifest monotonicity. We propose the use of Bayes factors to quantify the degree of support available in the data in favor of manifest monotonicity or against manifest monotonicity. Through the use of informative hypotheses, this procedure can also be used to determine the support for manifest monotonicity over substantively or statistically relevant alternatives to manifest monotonicity, rendering the procedure highly flexible. The performance of the procedure is evaluated using a simulation study, and the application of the procedure is illustrated using empirical data.

## Introduction

In item response theory (IRT) for dichotomously scored items, the assumption of latent monotonicity is shared by most parametric and nonparametric models. This assumption states that the probability of observing a positive response to an item is monotonically nondecreasing as a function of the latent variable, and plays an important role in obtaining the monotone likelihood-ratio property of the total score (Grayson, 1988; Hemker, Sijtsma, Molenaar, & Junker, 1997). The monotone likelihood-ratio property implies that the total score stochastically orders respondents on the latent variable, and this ordinal level of measurement is crucial to most applications of IRT. Latent monotonicity also captures the idea that the items in a test measure the latent variable (Junker & Sijtsma, 2000). For these reasons, investigating whether the assumption of latent monotonicity holds is important and relevant for many applications of IRT.

Because the latent variable is unobservable, latent monotonicity can only be evaluated indirectly, by considering observable consequences of the assumption. Given the assumption of local independence, latent monotonicity implies monotonicity over a variety of manifest scores, such as a single item score (Mokken, 1971), the unweighted restscore (Rosenbaum, 1984; Junker & Sijtsma, 2000), and any other sum score that does not include the item under consideration. By testing whether monotonicity holds at the manifest level—manifest monotonicity for short—, given the assumption of local independence one can investigate whether latent monotonicity is violated. Tijmstra, Hessen, Van der Heijden, and Sijtsma (2013) showed how the property of manifest monotonicity can be evaluated for a variety of manifest scores using order-constrained statistical inference, resulting in a likelihood-ratio test that determines whether there is sufficient evidence to reject monotonicity for the manifest score. A violation of manifest monotonicity implies a violation of latent monotonicity, hence a significant test statistic results in the rejection of latent monotonicity. Alternative methods for investigating latent monotonicity exist which use a manifest score (see, e.g., Rosenbaum, 1984) or the set of observed item-score patterns (Scheiblechner, 2003). Other nonparametric approaches have been developed, which estimate the item response function (IRF), making use of binning (Molenaar & Sijtsma, 2000), kernel smoothing (Ramsay, 1991), or spline-fitting (Abrahamowicz & Ramsay, 1992). These methods use local statistical tests, and also confidence bands are used to assess manifest monotonicity.

The aforementioned approaches have in common that they use a null hypothesis that specifies a boundary case of manifest monotonicity, also known as the ‘least favorable null hypothesis’ (Silvapulle & Sen, 2005) that still corresponds to manifest monotonicity. This null hypothesis is tested against the alternative hypothesis that manifest monotonicity does not hold. The specific form of this null hypothesis differs for each of these approaches, but they all use the boundary case where there is no association between the item scores and hence where the item-response probabilities are unrelated to the manifest score. The rationale behind using this hypothesis is that it considers the boundary of the part of the parameter space that corresponds to manifest monotonicity; if manifest monotonicity cannot be rejected for those parameter values, the data are consistent with at least one point in the parameter space that corresponds to manifest monotonicity. However, since in test construction items are usually designed to measure one common attribute, this null hypothesis is highly implausible in most practical settings.

Although these approaches are theoretically sound, by using the least favorable null hypothesis they may have suboptimal power to detect violations of manifest monotonicity. That is, in controlling the Type I error rate and ensuring that it does not exceed the specified significance level and that latent monotonicity is not rejected if there is at least one point in the parameter subspace with which the data are consistent, these approaches may be erring on the conservative side and inflate the Type II error rate; that is, they may fail to accumulate enough evidence to correctly reject latent monotonicity. Failing to detect violations of latent monotonicity could lead to using an IRT model whose estimates cannot be trusted. Arguably, this could be worse than incorrectly concluding that latent monotonicity does not hold and not applying an IRT model. Thus, it is important that a test for latent monotonicity has sufficient power to detect violations.

Furthermore, the approaches discussed so far use the null hypothesis testing framework and aim at falsification. That is, the tests attempt to provide a ‘critical test’ for the model assumption to see whether the assumption is able to ‘survive’ this test. However, failing to reject an assumption does not imply that it actually holds, since a Type II error could have been made. Since model assumptions have to hold for the model to be valid, simply noting that the assumption has failed to be rejected does not suffice as justification for applying the model. A power analysis may help to some extent to indirectly assess the amount of support that the model assumption receives when it fails to be rejected. However, one could argue that a more direct way of assessing support in favor of the model assumption is needed if a decision needs to be made whether using the model would be justifiable. The discussed frequentist approaches do not provide this kind of confirmatory support.

It is with these goals of increasing the power and directly assessing the support in favor of monotonicity in mind that we will pursue a Bayesian approach to evaluating latent monotonicity. Many different Bayesian model comparison approaches are available (e.g., see Gelman, Carlin, Stern, & Rubin, 2004), but of special interest here is the approach that focuses on the Bayes factor (see Hoijtink, 2012; Kass & Raftery, 1995). Using this approach, different hypotheses may be compared without assigning special status to one of the hypotheses by labeling it as a ‘null hypothesis.’ Rather than attempting to reject this null hypothesis, one investigates which hypothesis receives the most support from the data. Also, rather than resulting in a dichotomous outcome to reject or retain the assumption of latent or manifest monotonicity, an approach that uses the Bayes factor quantifies the degree of support each hypothesis receives from the data. This approach provides researchers with more information about the plausibility of the different hypotheses and enables them to make an informed decision about the credibility of the assumption of latent monotonicity. Furthermore, a Bayes factor approach allows for more than just contrasting the hypothesis of manifest monotonicity with the general hypothesis that manifest monotonicity does not hold (Tijmstra et al., 2013). Rather, a wide variety of hypotheses that are relevant in the context of monotonicity can be compared, allowing for finer nuances than just accepting or rejecting monotonicity.

This article proposes a Bayesian approach to evaluating manifest monotonicity for dichotomous item scores, in line with the Bayesian informative hypothesis testing framework discussed by Hoijtink (2012). First, several hypotheses that are relevant for latent monotonicity are discussed. Second, following Hoijtink (2012), we discuss how Bayes factors can be used to evaluate informative hypotheses, and we propose a procedure for estimating the relevant Bayes factors using Gibbs sampling. Third, we discuss a simulation study in which the performance of the procedure is evaluated under varying conditions and compared to a null hypothesis testing procedure that evaluates the same hypotheses (Tijmstra et al., 2013). Fourth, we discuss an empirical example of the application of the proposed procedure. The article concludes with a discussion.

## Relevant Competing Hypotheses

For a test containing *k* dichotomous items, let $X_{i}$ denote the score on item *i*, with realization $x_{i} = 0, 1$ for a negative and positive score, respectively. Let $\theta$ denote the latent variable. Latent monotonicity specifies that the IRF, denoted by $P(X_{i} = 1|\theta )$, is nondecreasing in $\theta$ (Hambleton & Swaminathan, 1985). The manifest score, denoted by *Y* and with realization *y*, is defined (Tijmstra et al., 2013) as

1 $$\begin{aligned} Y = \sum _{i=1}^{k} c_{i}X_{i}, \end{aligned}$$

where $c_{1}, \ldots , c_{k}$ are binary item inclusion coefficients that are chosen by the researcher. For example, by choosing $c_{j} = 0$ and $c_{i} = 1$ for all $i \ne j$, one obtains the unweighted restscore for item *j*. Including item *j* in the manifest score may confound the results (Junker & Sijtsma, 2000). Instead of using the total score, one may consider using the unweighted restscore. Although other manifest scores could be considered, the restscore is a more reliable ordinal estimator of the latent variable than a manifest score that is based on fewer items, provided the items that are included in the restscore are of good quality. The proposed procedure can be applied regardless of the specific choice of the manifest score.

Let *h* denote the highest possible value of manifest score *Y*, to be be obtained by means of $h = \sum _{i=1}^{k}c_{i}$. Furthermore, let $\pi _{y} = P(X = 1|Y = y)$ for the item that is investigated, where subscript *j* is dropped for notational convenience. The hypothesis that manifest monotonicity over *Y* holds for a specific item corresponds to

$$\begin{aligned} \begin{array}{ll} H_\mathrm{{MM}}:&\pi _{0} \le \cdots \le \pi _{y} \le \cdots \le \pi _{h}. \end{array} \end{aligned}$$

$H_\mathrm{{MM}}$ corresponds to the null hypothesis in the order-constrained statistical inference framework discussed by Tijmstra et al. (2013), and can be contrasted with its negation, which is the hypothesis that there are manifest nonmonotonicities:

$$\begin{aligned} \begin{array}{lll} H_\mathrm{{NM}}:&\pi _{y} > \pi _{y + 1},&\text {for at least one value of }y. \end{array} \end{aligned}$$

Because these hypotheses are mutually exclusive and exhaustive, evaluating manifest monotonicity effectively boils down to choosing between $H_\mathrm{{MM}}$ and $H_\mathrm{{NM}}$. However, $H_\mathrm{{NM}}$ is quite general, and hence not very informative. That is, if one accepts $H_\mathrm{{NM}}$, then little can be said about the ordering of the conditional item probabilities $\pi _{0}, \ldots , \pi _{h}$, other than that their ordering is not completely monotone. Following the terminology of Hoijtink (2012), $H_\mathrm{{NM}}$ has a high complexity, or similarly, $H_\mathrm{{NM}}$ is relatively unspecific or uninformative.

In practical applications, it may be important to know to which extent manifest monotonicity holds, that is, the extent to which the ordering of the conditional item probabilities are similar to the ordering specified by manifest monotonicity. Items for which the two orderings are almost the same could be considered to be *essentially* monotone, and might still be of practical use. For example, one could define essential monotonicity as a less restrictive version of manifest monotonicity, allowing for local violations of manifest monotonicity ($\pi _{y} > \pi _{y + 1}$ for some *y*) as long as these violations occur only between adjacent values of *Y*. If one would consider including such essentially monotone items in a test, one should carefully consider whether this does not threaten the stochastic ordering of persons. The extent to which the stochastic ordering of persons based on the total score is robust against inclusions of not fully monotone items has not been studied extensively (but see Van der Ark, 2005), but in case the scale is robust against these kind of violations essentially monotone items could provide a useful addition to a test. Hence, finding out whether items are strictly monotone, essentially monotone, or nonmonotone can be of interest to for example test constructors.

The hypothesis that a form of ‘essential monotonicity’ holds for a specific item may be formulated as

$$\begin{aligned} \begin{array}{lll} H_\mathrm{{EM}}: &{}\pi _{0} \le min \{\pi _{2},\pi _{3}\},\\ &{}\pi _{1} \le min \{\pi _{3},\pi _{4}\},\\ &{}\quad \vdots \\ &{}\pi _{h-3} \le min \{\pi _{h-1},\pi _{h}\},\\ &{}\pi _{h-2} \le \pi _{h}. \end{array} \end{aligned}$$

In this formulation, essential monotonicity is violated as soon as for some *y*, $\pi _{y} > \pi _{y + d}$ for some $d \in \{2, \ldots , h - y\}$. More liberal versions of essential monotonicity can be obtained by letting $d \in \{e, \dots , h - y\}$, where $e > 2$. The larger the value that is chosen for *e*, the less restrictive and the less informative $H_\mathrm{{EM}}$ becomes, up to the point where $H_\mathrm{{EM}}$ hardly captures monotonicity anymore. In addition to its potential substantive relevance, investigating essential monotonicity helps to increase the power to detect small violations of manifest monotonicity. This potential increase in power is due to $H_\mathrm{{EM}}$ placing more restrictions on the conditional item probabilities than $H_\mathrm{{NM}}$; hence, $H_\mathrm{{EM}}$ is more specific.

Another interesting alternative to $H_\mathrm{{MM}}$ is the postulation of a ceiling or a floor effect, formulated in $H_\mathrm{{C}}$ and $H_\mathrm{{F}}$ as, respectively:

$$\begin{aligned} \begin{array}{ll} H_\mathrm{{C}}: &{}\pi _{0} \le \ldots \le \pi _{c}; \{\pi _{c + 1}, \ldots , \pi _{h}\}, \text {and}\\ H_\mathrm{{F}}: &{}\{\pi _{0}, \ldots , \pi _{f - 1}\}; \pi _{f} \le \ldots \le \pi _{h},\\ \end{array} \end{aligned}$$

where *c* denotes the ‘ceiling-value’ and *f* the ‘floor-value’ of the manifest score. Both $H_\mathrm{{C}}$ and $H_\mathrm{{F}}$ leave the ordering of some of the conditional item probabilities open, thus allowing for nonmonotonicities above ($H_\mathrm{{C}}$) or below ($H_\mathrm{{F}}$) a particular value of *Y*. This weaker form of monotonicity may be of interest for selection or testing purposes, for example, when the main goal of a test is to distinguish respondents on either the low or on the high end of the distribution but not necessarily across the entire scale. In addition, the hypotheses may be useful in the context of exam items, where the possibility of providing the desired answer may decrease for examinees at the high end of the scale, or in the context of multiple choice items where some distractors may fail for low-ability examinees.

Like $H_\mathrm{{EM}}$, $H_\mathrm{{C}}$ and $H_\mathrm{{F}}$ are more restrictive than $H_\mathrm{{NM}}$, which could result in increased power to detect specific violations of monotonicity. Focussing on these specific kinds of deviations from monotonicity could result in a higher power to detect these violations, and could also have substantive relevance in some applications of IRT. The section dealing with the empirical example illustrates the value of considering such informative alternative hypotheses in addition to considering $H_\mathrm{{NM}}$. In order to be able to evaluate the hypotheses, we first discuss the use of Bayes factors.

## Bayes Factors

The relative support for either of two competing hypotheses can be quantified using the Bayes factor (Jeffreys, 1961; Kass & Raftery, 1995). The Bayes factor balances the fit of the different hypotheses against their complexity. To determine the fit and the complexity of a hypothesis $H_\mathrm{{Z}}$ imposing order constraints on $\pi _{0}, \ldots , \pi _{h}$, a prior distribution of $\varvec{\pi }=(\pi _{1}, \ldots , \pi _{h})$ needs to be specified, and the posterior distribution of $\varvec{\pi }$ after observing the data also needs to be determined.

In order to ensure that every ordering of $\pi _{0}, \ldots , \pi _{h}$ is equally likely a priori (Hoijtink, 2012), one can specify the prior distribution to be

2 $$\begin{aligned} h(\varvec{\pi }) = \prod _{y=0}^{h}Beta(\pi _{y};1,1) = 1. \end{aligned}$$

This prior distribution does not favor any specific ordering of $\pi _{0}, \ldots , \pi _{h}$, and for each $\pi _{y}$ assigns equal probability to all values between 0 and 1; hence, it can be considered to be uninformative (Lynch, 2007). Since under the prior distribution in Equation 2 every ordering is a priori considered to be equally likely, the complexity of every inequality-constrained hypothesis can in principle be determined analytically (Hoijtink, 2012).

Assuming the scores on the item to be binomially distributed for each value of the manifest score, the likelihood of the data corresponds to

3 $$\begin{aligned} f(\mathbf{X}|\varvec{\pi }) = \prod _{y=0}^{h}\pi _{y}^{s_{y}}(1 - \pi _{y})^{n_{y}-s_{y}}, \end{aligned}$$

where $\mathbf{X}$ denotes the vector containing the scores on the item in question, $n_{y}$ denotes the number of respondents with manifest score *y*, and $s_{y}$ denotes the number of respondents with manifest score *y* for whom $X_j = 1$. The posterior distribution of the conditional item probabilities is proportional to the product of the likelihood and the prior distribution, and corresponds to

4 $$\begin{aligned} g(\varvec{\pi }|\mathbf{X}) = \prod _{y=0}^{h}Beta(\pi _{y};s_{y} + 1, n_{y} - s_{y} + 1). \end{aligned}$$

Following the framework proposed by Hoijtink (2012), the complexity $c_\mathrm{{Z}}$ of a hypothesis $H_\mathrm{{Z}}$ can be defined as the proportion of the prior distribution of $\varvec{\pi }$ that is in accordance with this hypothesis. Thus, for a hypothesis $H_\mathrm{{Z}}$,

5 $$\begin{aligned} c_{Z} = \frac{\int h(\varvec{\pi })\mathcal {I}_{\varvec{\pi }\in \mathcal {H}_\mathrm{{Z}}}d\varvec{\pi }}{\int h(\varvec{\pi })d\varvec{\pi }}= \int h(\varvec{\pi })\mathcal {I}_{\varvec{\pi }\in \mathcal {H}_\mathrm{{Z}}}d\varvec{\pi }, \end{aligned}$$

where $\mathcal {H}_\mathrm{{Z}}$ denotes the infinite set that contains all vectors $\varvec{\pi }$ for which $H_\mathrm{{Z}}$ is fulfilled, and where $\mathcal {I}_{\varvec{\pi }\in \mathcal {H}_\mathrm{{Z}}}$ is an indicator function that equals 1 if $\varvec{\pi } \in \mathcal {H}_\mathrm{{Z}}$, and 0 otherwise. Thus, the complexity of a hypothesis such as $H_\mathrm{{MM}}$ corresponds to the probability of obtaining a set of values for $\varvec{\pi }$ that match the constraints specified by $H_\mathrm{{MM}}$ if we were to randomly draw values from the prior distribution of $\varvec{\pi }$.

In a similar vein, the posterior fit $f_\mathrm{{Z}}$ of hypothesis $H_\mathrm{{Z}}$ to the data can be defined as the proportion of the posterior distribution of $\varvec{\pi }$ that is in accordance with that hypothesis (Hoijtink, 2012), and corresponds to

6 $$\begin{aligned} f_\mathrm{{Z}} = \frac{\int g(\varvec{\pi }|\mathbf{X})I_{\varvec{\pi }\in \mathcal {H}_\mathrm{{Z}}}d\varvec{\pi }}{\int g(\varvec{\pi }|\mathbf{X})d\varvec{\pi }} = \int g(\varvec{\pi }|\mathbf{X})I_{\varvec{\pi }\in \mathcal {H}_\mathrm{{Z}}}d\varvec{\pi }. \end{aligned}$$

By comparing the fit of a hypothesis with its complexity, one can determine the extent to which the data provide evidence in favor of or against the hypothesis. The ratio $\frac{f}{c}$ quantifies how much more likely the hypothesis has become after observing the data, and hence, it reflects the amount of support that the hypothesis receives from the data (Kass & Raftery, 1995). The Bayes factor comparing two competing hypotheses that specify order constraints for $\varvec{\pi }$ can be calculated by taking the ratio of $\frac{f}{c}$ of the two hypotheses (Hoijtink, 2012). Thus, the Bayes factor does not simply contrast the fit of two hypotheses to the data, but rewards hypotheses that are more specific by taking their complexity into account.

### Bayes Factors and Monotonicity

With regard to manifest monotonicity, the simplest comparison that can be made is between $H_\mathrm{{MM}}$ and the unconstrained alternative $H_\mathrm{{U}}:\{\pi _{0}, \ldots , \pi _{h}\}$. The corresponding Bayes factor (*BF*) can be computed by means of

$$\begin{aligned} BF_\mathrm{{MM,U}} = \frac{\frac{f_\mathrm{{MM}}}{c_\mathrm{{MM}}}}{\frac{f_\mathrm{{U}}}{c_\mathrm{{U}}}} = \frac{f_\mathrm{{MM}}}{c_\mathrm{{MM}}}. \end{aligned}$$

Here, because $H_\mathrm{{U}}$ does not restrict $\varvec{\pi }$ and hence $f_\mathrm{{U}} = c_\mathrm{{U}} = 1$, $\frac{f_\mathrm{{U}}}{c_\mathrm{{U}}}$ drops out of the equation. If $BF_\mathrm{{MM,U}} > 1$, the data provide support for $H_\mathrm{{MM}}$, whereas $BF_\mathrm{{MM,U}} < 1$ indicates that the data do not support the hypothesis of manifest monotonicity.

Since $H_\mathrm{{U}}$ incorporates $H_\mathrm{{MM}}$, contrasting $H_\mathrm{{MM}}$ with $H_\mathrm{{U}}$ is not very informative. In order to evaluate $H_\mathrm{{MM}}$, this hypothesis should be contrasted with a competing hypothesis. For example, one may contrast $H_\mathrm{{MM}}$ with its complement $H_\mathrm{{NM}}$, which posits that the conditional probabilities do not increase monotonically:

$$\begin{aligned} BF_\mathrm{{MM,NM}} = \frac{f_\mathrm{{MM}}c_\mathrm{{NM}}}{f_\mathrm{{NM}}c_\mathrm{{MM}}} = \frac{f_\mathrm{{MM}}\left( 1 - c_\mathrm{{MM}}\right) }{\left( 1 - f_\mathrm{{MM}}\right) c_\mathrm{{MM}}}. \end{aligned}$$

Thus, $BF_\mathrm{{MM,NM}}$ quantifies the amount of support that $H_\mathrm{{MM}}$ receives from the data when contrasted with its complement. The comparison of $H_\mathrm{{MM}}$ and $H_\mathrm{{NM}}$ provides useful information about the general support for the hypothesis that the conditional item probabilities are ordered in accordance with manifest monotonicity.

By only considering a subset of the orderings that $H_\mathrm{{NM}}$ allows, manifest monotonicity can be contrasted with more specific alternatives. If realistic alternative hypotheses are selected, the power to detect violations of manifest monotonicity may increase, since these alternatives may receive more support from the data than the uninformative $H_\mathrm{{NM}}$. For example, one may consider contrasting $H_\mathrm{{MM}}$ with $H_\mathrm{{EM}}$, thereby excluding all orderings that deviate strongly from monotonicity. Considering $H_\mathrm{{EM}}$ can be particularly useful when much is known about a test and possible deviations from monotonicity are expected to be modest. In order to construct hypotheses that are mutually exclusive, one can define $H_\mathrm{{EM}'}$ as $H_\mathrm{{EM}}$ with the constraint that $H_\mathrm{{MM}}$ does not hold. For this comparison, one obtains

$$\begin{aligned} BF_\mathrm{{MM,EM}'} = \frac{f_\mathrm{{MM}}c_\mathrm{{EM}'}}{f_\mathrm{{EM}'}c_\mathrm{{MM}}} = \frac{f_\mathrm{{MM}}\left( c_\mathrm{{EM}} - c_\mathrm{{MM}}\right) }{\left( f_\mathrm{{EM}} - f_\mathrm{{MM}}\right) c_\mathrm{{MM}}}. \end{aligned}$$

Similarly, one can contrast $H_\mathrm{{MM}}$ with $H_\mathrm{{C}'}$ or $H_\mathrm{{F}'}$, where $H_\mathrm{{C}'}$ or $H_\mathrm{{F}'}$ are obtained from $H_\mathrm{{C}}$ and $H_\mathrm{{F}}$ by adding the constraint that $H_\mathrm{{MM}}$ does not hold. The Bayes factors $BF_\mathrm{{MM,C}'}$ and $BF_\mathrm{{MM,F}'}$ indicate whether there is reason to suspect that monotonicity is violated at the high end or the low end of the manifest scale, respectively.

### Estimating the Bayes Factors

The estimation of the Bayes factor requires one to obtain the fit and the complexity of the two hypotheses of interest. Under the uninformative prior distribution of $\varvec{\pi }$ in Equation 2 (and under any exchangeable prior), each ordering of the conditional item probabilities is equally likely, and the complexity of any hypothesis $H_\mathrm{{Z}}$ about the ordering of these conditional item probabilities can be obtained by means of

$$\begin{aligned} c_\mathrm{{Z},h} = \frac{O_\mathrm{{Z},h}}{(h+1)!}, \end{aligned}$$

where $O_{\mathrm{{Z}},h}$ denotes the number of possible orderings of the conditional item probabilities that are allowed by $H_\mathrm{{Z}}$, given that the highest possible value on the manifest score equals *h*.

Thus, it follows that $O_{MM,h} = 1$, $O_{C,h} = \frac{(h + 1)!}{(h + 1 - c)!}$ and $O_{F,h} = \frac{(h + 1)!}{(f + 1)!}$. The number of orderings that essential monotonicity allows is a number from the Fibonacci sequence. That is, $O_{\mathrm{EM},h} = \text {Fib}_{h + 3}$, where $\mathbf{Fib} = \{0, 1, 1, 2, 3, 5, 8, 13,\ldots \}$. Because the constraints in $H_\mathrm{{EM}}$ specify that conditional probabilities two score units apart cannot decrease, increasing *h* by 1 increases the number of acceptable orderings by $O_{\mathrm{EM},h-1}$. That is, when *h* increases by 1 (i.e., an item is added to the test), the highest possible manifest score becomes $h + 1$, and there are two types of orderings possible that are allowed by $H_{\mathrm{EM}}$: Orderings where $\pi _{h} \le \pi _{h+1}$, of which there are $O_{\mathrm{EM},h}$ in total, and orderings where $\pi _{h-1} \le \pi _{h+1} < \pi _{h}$, of which there are $O_{\mathrm{EM},h - 1}$. Thus, for any $h > 0$, $O_{\mathrm{EM},h+1} = O_{\mathrm{EM},h} + O_{\mathrm{EM},h-1}$, resulting in the Fibonacci sequence. The complexities of $H_\mathrm{{EM}'}$, $H_\mathrm{{C}'}$, and $H_\mathrm{{F}'}$ can be obtained by subtracting 1 from $O_{\mathrm{EM},h}$, $O_{\mathrm{C},h}$ and $O_{\mathrm{F},h}$, respectively.

Analytically determining the fit of the hypotheses is not straightforward. Instead of exact integration in Equation 6, a Gibbs sampling procedure can be used to approximate the proportion of the posterior that falls within the specified part of the parameter space. This procedure enables one to repeatedly sample values of $\varvec{\pi }$ from its posterior distribution, thus allowing one to approximate the posterior distribution to any degree of precision and hence, making it possible to approximate the value of $f_\mathrm{{Z}}$ for any $H_\mathrm{{Z}}$. However, since $f_\mathrm{{Z}}$ may be extremely small for large values of *h*, estimating $f_\mathrm{{Z}}$ simply by counting the proportion of draws from the posterior distribution of $\varvec{\pi }$ that are in accordance with the constraints specified in $H_\mathrm{{Z}}$ does not necessarily result in an accurate estimate of $f_\mathrm{{Z}}$, unless one evaluates an excessively large number of draws.

A computationally less demanding approach is to sequentially evaluate the individual constraints specified in $H_\mathrm{{Z}}$. This can be done by decomposing the Bayes factor of a hypothesis $H_\mathrm{{Z}}$ with *w* constraints against $H_\mathrm{{U}}$ into *w* Bayes factors (Mulder et al., 2009) as

7 $$\begin{aligned} BF_{Z,U}= & {} BF_{1,\mathrm{{U}}} \times BF_{2,1} \times \ldots \times BF_{v,v-1} \times \ldots \times BF_{w,w-1}\nonumber \\= & {} \frac{f_{1|\mathrm{{U}}}}{c_{1|\mathrm{{U}}}} \times \frac{f_{2|1}}{c_{2|1}} \times \ldots \times \frac{f_{v|v-1}}{c_{v|v-1}} \times \ldots \times \frac{f_{w|w-1}}{c_{w|w-1}}\nonumber \\= & {} \frac{f_{1|\mathrm{{U}}} \times f_{2|1} \times \ldots \times f_{v|v-1} \times \ldots \times f_{w|w-1}}{c_{\mathrm{{Z}}}}. \end{aligned}$$

Here, $BF_{1,\mathrm {U}}$ is the Bayes factor comparing the hypothesis that the first order constraint holds ($H_{1}$) with the unconstrained hypothesis ($H_\mathrm{{U}}$), and $BF_{v,v-1}$ is the Bayes factor comparing the hypothesis that the first *v* order constraints hold ($H_{v}$) with the hypothesis that the first $v-1$ constraints hold ($H_{v-1}$). Furthermore, $f_{v|v-1}$ is the fit of $H_{v}$ conditional on the assumption that $H_{v-1}$ holds. For each hypothesis $H_{v}$, this conditional fit measure $f_{v|v-1}$ can be estimated using a Gibbs sampling procedure (see e.g. Geman & Geman, 1984) that draws values from the joint posterior distribution of $\varvec{\pi }$ under the $v-1$ constraints of $H_{v-1}$, that is,

8 $$\begin{aligned} g\big (\varvec{\pi }|\mathbf{X}; \varvec{\pi }\in \mathcal {H}_{v-1}\big ) \propto \prod _{y=0}^{h}Beta\left( \pi _{y};s_{y} + 1, n_{y} - s_{y} + 1\right) \mathcal {I}_{\varvec{\pi }\in \mathcal {H}_{v-1}}. \end{aligned}$$

To sample from this multivariate distribution, in each iteration of the Gibbs sampler we subsequently sample from the individual full conditional posterior distributions of each $\pi _{y}$, given the current values of all other parameters. Equation 8 implies that the full conditional posterior distribution of each $\pi _{y}$ is either a truncated beta distribution if $\pi _{y}$ is constrained by $H_{v-1}$, or a regular beta distribution otherwise. After allowing for a burn-in period (e.g., after discarding the first 5000 draws), these draws result in an approximation of the joint posterior distribution $g(\varvec{\pi }|\mathbf{X}; \varvec{\pi }\in \mathcal {H}_{v-1})$ that can be used to estimate $f_{v|v-1}$ (e.g., using 10,000 draws). By sequentially applying this Gibbs sampler to estimate $f_{1|\mathrm{u}}, \ldots , f_{w|w-1}$, one can approximate $f_{Z}$. This procedure enables the approximation of the fit of any hypothesis imposing order constraints on $\varvec{\pi }$.

### Using the Bayes Factor

The Bayes factor can be obtained for any pair of order-constrained hypotheses about the conditional item probabilities. The procedure we discussed has been implemented as a function in R (R Core Team, 2014) that can be used to evaluate manifest monotonicity, by contrasting $H_\mathrm{{MM}}$ with $H_\mathrm{{NM}}$ as well as $H_\mathrm{{EM}'}$. The test function is available on request from the first author.

Kass and Raftery (1995) provide general guidelines for the interpretation of Bayes factors (also, see Jeffreys, 1961): If $\frac{1}{3} < BF < 3$, there is little support for either hypothesis; if $3 \le BF < 20$ or $\frac{1}{20} < BF \le \frac{1}{3}$ there is some support in favor of the first hypothesis or the second hypothesis, respectively; if $BF\ge 20$ or $BF\le \frac{1}{20}$, there is strong support in favor of the first hypothesis or the second hypothesis, respectively.

One might consider accepting latent monotonicity only if there is strong support for $H_\mathrm{{MM}}$ over $H_\mathrm{{NM}}$ ($BF_\mathrm{{MM,NM}} \ge 20$), and keep the item that was evaluated in the test. If the aim is falsification, one could decide to reject latent monotonicity when strong support is found against manifest monotonicity relative to its complement $H_\mathrm{{NM}}$ ($BF_\mathrm{{MM,NM}} \le \frac{1}{20}$). However, this could result in keeping malfunctioning items in a test simply because the evidence was inconclusive. Alternatively, we propose to only retain items for which $BF_\mathrm{{MM,NM}} \ge 20$.

**Fig. 1:**
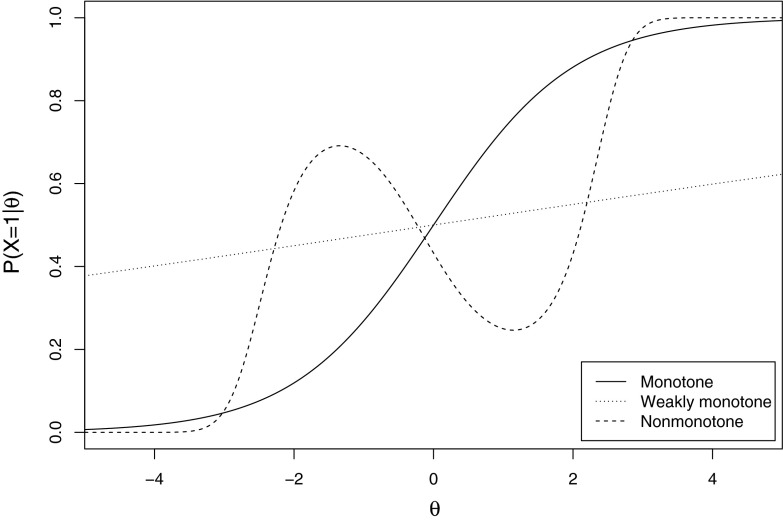
The item response functions of the three items that were analyzed.

One may consider to let the consequences of the comparison of $H_\mathrm{{EM}'}$ and $H_\mathrm{{MM}}$ depend on the particular circumstances of the application at hand. For some low-stakes settings, it may be sufficient that an item shows an overall positive trend (i.e., it is essentially monotone), but for high-stakes tests, one could demand that even small violations of latent monotonicity as captured by $H_\mathrm{{EM}'}$ are unacceptable and only retain items for which there is at least some positive evidence (i.e., $BF_\mathrm{{MM,EM}'} \ge 3$) that $H_\mathrm{{MM}}$ rather than $H_\mathrm{{EM}'}$ holds.

## Simulation Study

### Method

To facilitate the comparison of the proposed procedure to that of existing methods for evaluating latent monotonicity, conditions similar to those discussed by Tijmstra et al. (2013) were used in a simulation study. In this way, the decisions that would be made using the proposed method could be compared to those that would be made using the order-constrained null hypothesis test discussed by Tijmstra et al. (2013). The procedure was used to assess manifest monotonicity for three items, corresponding to three different relevant scenarios: A ‘normal’ item with a monotone IRF that discriminates well, a weakly discriminating item with a monotone but nearly flat IRF, and an item with a locally nonmonotone IRF (Figure 1). For convenience, we label these three items ‘monotone item’, ‘weak item’, and ‘nonmonotone item’, respectively. The monotone item represents a typical desirable item that provides a useful contribution to the test, the weak item represents an item that contributes little to the reliable ordering of persons but does not violate latent monotonicity, and the nonmonotone item represents a problematic item that should not be included in the test.

The IRFs of the monotone item and the weak item were two-parameter logistic with difficulty parameters equal to 0 and discrimination parameters equal to 1 and .1, respectively. For the nonmonotone item, a locally nonmonotone IRF was obtained using a polynomial extension of the two-parameter logistic model previously used by Tijmstra et al. (2013),

$$\begin{aligned} P(X_{i}=1|\theta ) = \frac{\exp \left( \alpha _{1i}\big (\theta - \beta _{1i}\big ) + \alpha _{2i}\big (\theta - \beta _{2i}\big )^{2} + \alpha _{3i}\big (\theta - \beta _{3i}\big )^{3}\right) }{1 + \exp \left( \alpha _{1i}\big (\theta - \beta _{1i}\big ) + \alpha _{2i}\big (\theta - \beta _{2i}\big )^{2} + \alpha _{3i}\big (\theta - \beta _{3i}\big )^{3}\right) }, \end{aligned}$$

where $\beta _{1i}$, $\beta _{2i}$, and $\beta _{3i}$ influence the difficulty of the item and $\alpha _{1i}$, $\alpha _{2i}$, and $\alpha _{3i}$ influence the slope of the IRF. Following Tijmstra et al. (2013), we chose $\alpha _{1i}$, $\alpha _{2i}$ and $\alpha _{3i}$ equal to 1, 1.2, and 0.25, respectively, and $\beta _{1i}$, $\beta _{2i}$ and $\beta _{3i}$ equal to 2.5, 1.6, and 1.5, respectively.

**Fig. 2:**
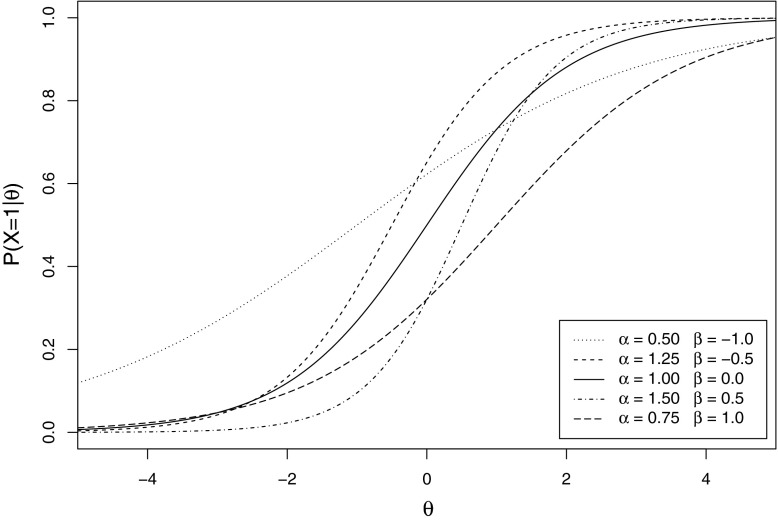
The item response functions of the five monotone items, based on the two-parameter logistic model. The discrimination and difficulty parameters are denoted by $\alpha$ and $\beta$, respectively.

Test length was varied by considering manifest scores obtained based on 5, 10, and 20 dichotomous monotone items. The items included in the manifest score were specified using the two-parameter logistic model; the IRFs are displayed in Figure 2. Five different IRFs were specified, with difficulty parameters $\{-1, -0.5, 0, 0.5, 1\}$ and discrimination parameters $\{0.5, 1.25, 1, 1.25, 1.50\}$, matching the design of Tijmstra et al. (2013). For manifest scores based on 10 and 20 items, two and four duplicates of the 5-item set were used, respectively. Sample sizes (*n*) of 100, 200, 500, and 1000 were used to study the effect sample size had on the values of the Bayes factors and the resulting decisions about manifest monotonicity based on the proposed guidelines.

For each design condition, 1000 replications were generated. For each replication, *n* values of the latent variable were drawn from a standard normal distribution, and subsequently item scores were generated, yielding data matrices for the item of interest (monotone, weak, or nonmonotone) and the 5, 10, or 20 items that were used to compute the manifest score. Next, the Bayesian procedure was applied to the generated data, using 5000 iterations for the burn-in period of the Gibbs sampler and the subsequent 10,000 iterations to approximate the posterior distribution $g(\varvec{\pi }|\mathbf{X}; \varvec{\pi }\in \mathcal {H}_{v-1})$ for each order constraint *v*, as detailed in Equation 7. This way, the Bayes factors of $H_\mathrm{{MM}}$ versus $H_\mathrm{{NM}}$ and of $H_\mathrm{{MM}}$ versus $H_\mathrm{{EM}'}$ were obtained for each replication.

**Table 1:** Proportion of rejections of latent monotonicity for the nonmonotone item using the Bayes factor procedure (1000 replications) and the order-constrained NHST procedure, for varying sample size (rows) and test length (columns).

	Bayes factor						NHST		
	$BF_\mathrm{{MM,NM}} \le \frac{1}{20}$			$BF_\mathrm{{MM,NM}} \ge 20$			$p < .05$		
*n*	5	10	20	5	10	20	5	10	20
100	.702	.833	.911	.000	.001	.000	.334	.404	.457
200	.902	.958	.981	.000	.001	.000	.553	.645	.652
500	*	*	*	*	*	*	.931	.800	.842

* Means that computational limitations prohibited computation of entries.

### Results

For the nonmonotone item, Table 1 reports the proportion of replications in which strong support is found against manifest monotonicity relative to its complement ($BF_\mathrm{{MM,NM}} \le \frac{1}{20}$), thus leading to a rejection of latent monotonicity. The results show that also for small samples the proposed procedure had a high power to correctly reject latent monotonicity; except for $k = 5$ and $n = 100$, the observed power levels exceeded .80 for all other conditions. The evidence against latent monotonicity increased quickly as sample size increased. For $n \ge 500$, some of the 1000 replications encountered difficulties with the estimation of the Bayes factor (empty cells in Table 1), as the constraints were so unlikely that the estimation of some of the full conditional posteriors in Equation 8 became unfeasible. Consequently, the Bayes factor could not be estimated for every replication in these conditions. This problem can only occur if there is overwhelming evidence against $H_\mathrm{{MM}}$, and only happens when the estimate of the Bayes factor approximately equals 0, as is the case when $n \ge 500$. Table 1 also shows that in at most 0.1 % of the replications strong support was found for manifest monotonicity. Thus, if one uses a strict guideline and only retains items for which $BF \ge 20$, items like the nonmonotone item will almost always be removed successfully.

Table 1 compares the power of the Bayesian procedure with Tijmstra et al.’s (2013) procedure based on the null hypothesis statistical testing (NHST) framework. The table presents the results obtained by Tijmstra et al. (2013) and compares them with the Bayesian result obtained under the same conditions. The Bayesian procedure outperformed the null hypothesis test, where for the latter acceptable power levels were found only for large sample sizes ($n = 500$). Unlike the NHST procedure, the Bayes factor procedure shows a marked gain in power as test length increased.

**Table 2:** Proportion of replications strongly agreeing or disagreeing with $H_\mathrm{{MM}}$ when contrasted with $H_\mathrm{{NM}}$ for the monotone item and the weak item (1000 replications), for varying sample size (rows) and test length (columns).

	Monotone item			Weak item		
*n*	5	10	20	5	10	20
	Strong support for $H_\mathrm{{MM}}$ over $H_\mathrm{{NM}}$					
100	.583	.841	.936	.039	.122	.176
200	.858	.965	.995	.057	.206	.324
500	.981	.998	1.000	.107	.315	.500
1000	.996	1.000	.999	.137	.406	.640
	Strong support for $H_\mathrm{{NM}}$ over $H_\mathrm{{MM}}$					
100	.003	.005	.004	.115	.194	.246
200	.000	.000	.002	.112	.140	.198
500	.000	.000	.000	.066	.114	.127
1000	.000	.000	.001	.041	.064	.079

Table 2 shows the results for the monotone item and the weak item when contrasting manifest monotonicity with its complement. For the monotone item, the proportion of replications where $BF_\mathrm{{MM,NM}} \ge 20$, indicating strong support for manifest monotonicity, exceeded .80 for most conditions, except for $n = 100$ and $k = 5$. The proportion of replications providing strong support against manifest monotonicity ($BF_\mathrm{{MM,NM}} \le \frac{1}{20}$) was always close to 0. As test length and sample size increased, the proportion of replications providing support for manifest monotonicity approached 1. Thus, in almost all but the most unfavorable conditions the procedure consistently indicated that manifest monotonicity held for the monotone item, and the monotone item had a high probability of correctly passing the first test of the procedure.

Table 2 also shows the results for the weak item. Compared to the monotone item, the proportion of replications providing strong support for manifest monotonicity was considerably smaller for the weak item in all conditions, especially for smaller sample sizes ($n = 100, 200$) and shorter tests ($k = 5, 10$). As *n* or *k* increased, the procedure more often found strong support for manifest monotonicity relative to its complement. For longer tests ($k = 20$) and for smaller sample sizes ($n < 500$), the proportion of replications showing strong support against manifest monotonicity was relatively large, up to .246 for $k = 20$ and $n = 100$. Even though one may expect occasional rejections of manifest monotonicity for weak items such as this one, the results may be considered surprising. Further study showed that the results are due to low-score and high-score groups having few observations in these conditions. When data are sparse, the uniform prior is relatively influential and pushes the estimates of the conditional probabilities toward .5. As a result, some replications result in $BF\le \frac{1}{20}$. For the monotone item, the evidence in favor of monotonicity was much stronger, resulting almost always in $BF \ge 20$ despite sparse data in some score groups.

The second part of the procedure contrasted $H_\mathrm{{MM}}$ with $H_\mathrm{{EM}'}$. Since it is more difficult to distinguish between $H_\mathrm{{MM}}$ and $H_\mathrm{{EM}'}$, we focused on the results suggesting at least some support in favor of one of the hypotheses ($BF \ge 3$ or $BF \le \frac{1}{3}$) rather than requiring strong support. Table 3 shows that for the monotone item, the proportion of replications providing support for manifest monotonicity relative to essential monotonicity varied greatly depending on test length and sample size. The proportion of cases where $H_\mathrm{{MM}}$ was correctly supported increased strongly as the sample size increased.

As test length increases, it is more difficult to distinguish the two hypotheses for the monotone item; see the relatively low proportion of cases with support for $H_\mathrm{{MM}}$ when $k = 20$ (Table 3). The explanation is that as test length increases, the differences in the mean ability of neighboring score groups grow smaller. Moreover, increasing test length given fixed *n* results in fewer observations per score group and less accurate estimates per group, especially for the extreme score groups. As a result of data sparsity, the estimates of the conditional probabilities in the extreme score groups may be strongly biased toward .5 because of the influence of the uniform prior. This means that for the extreme score groups the estimated conditional probabilities often show a decrease across the first and the last couple of score groups, even though the population conditional probabilities are strictly monotone. These different influences together impair finding evidence for a strictly monotone ordering relative to an essentially monotone ordering when *k* is large and *n* is small. As *n* increases, data sparsity becomes rare, and support for $H_\mathrm{{MM}}$ relative to $H_\mathrm{{EM'}}$ is found more frequently.

For the weak item, Table 3 shows that for short tests ($k \le 10$), the proportion of replications providing support for manifest monotonicity relative to essential monotonicity was small, even for $n = 1000$. This finding is in contrast with the results for the monotone item, where for $k \le 10$ and $n = 1000$ more than 80 % of replications showed support for monotonicity. However, for $k = 20$ the differences between the results for the weak item and the monotone item were less extreme and less clear. For longer tests ($k = 20$), the proportion of replications providing support for manifest monotonicity for the weak item increased slowly as *n* increased, up to .353 for $n = 1000$.

**Table 3:** Proportion of cases agreeing or disagreeing with $H_\mathrm{{MM}}$ when contrasted with $H_\mathrm{{EM}'}$ for the items with a monotone and a flat IRF (1000 replications), for varying sample size (rows) and test length (columns).

	Monotone item			Weak item		
*n*	5	10	20	5	10	20
	Support for $H_\mathrm{{MM}}$ over $H_\mathrm{{EM}'}$					
100	.223	.000	.015	.011	.007	.081
200	.495	.104	.032	.011	.009	.135
500	.819	.489	.234	.015	.013	.260
1000	.953	.815	.519	.024	.019	.353
	Support for $H_\mathrm{{EM}'}$ over $H_\mathrm{{MM}}$					
100	.039	.029	.006	.053	.036	.044
200	.026	.042	.003	.041	.033	.047
500	.003	.022	.013	.037	.021	.044
1000	.003	.008	.020	.024	.035	.036

## Empirical Example

The procedure was applied to evaluate manifest monotonicity for each item from a set of eleven four-option multiple-choice items measuring reading comprehension in sixth grade, primary school students. Data were obtained as part of a larger pilot study, and dichotomously scored responses to these items were available from 773 Dutch students. Because there was no a priori reason to exclude any item from the test, the unweighted restscore was used as the manifest score across which monotonicity was evaluated. For each of the items, the Bayes factors contrasting $H_\mathrm{{MM}}$ with $H_\mathrm{{NM}}$ and $H_\mathrm{{MM}}$ with $H_\mathrm{{EM}'}$ were estimated. Each Bayes factor was obtained through the decomposition in Equation 7, where each decomposed Bayes factor was estimated based on 10,000 draws from the corresponding joint posterior distribution (obtained after a burn-in period of 5000 iterations).

**Table 4:** Conditional proportions $p_{y}$ and Bayes factors for the eleven reading comprehension items.

Item	$p_{0}$	$p_{1}$	$p_{2}$	$p_{3}$	$p_{4}$	$p_{5}$	$p_{6}$	$p_{7}$	$p_{8}$	$p_{9}$	$p_{10}$	$BF_\mathrm{{MM,NM}}$	$BF_\mathrm{{MM,EM}'}$
1	n.a.	.50	.25	.67	.50	.23	.27	.20	.29	.26	.45	.001	.451
2	n.a.	.00	.33	.88	.85	.79	.88	.94	.95	.98	.90	2715	3.48
3	n.a.	.00	.33	.67	.64	.69	.84	.86	.92	.91	.90	14,701	5.27
4	n.a.	.67	.00	.57	.80	.91	.98	.98	.99	1.00	1.00	1543	1.67
5	n.a.	.00	.00	.25	.57	.61	.78	.84	.88	.92	1.00	90,189	8.57
6	n.a.	.50	.40	.90	.92	.81	.92	.95	.97	.98	1.00	3264	2.30
7	n.a.	.50	.25	.75	.82	.83	.93	.92	.95	.96	1.00	11,403	2.71
8	1.00	1.00	.00	.25	.00	.06	.15	.14	.18	.17	.35	.006	1.70
9	n.a.	.50	.00	.40	.57	.58	.74	.78	.87	.84	.90	6093	2.06
10	n.a.	.00	.00	.00	.21	.16	.23	.19	.22	.20	.41	46.7	1.78
11	n.a.	.50	.25	.80	.89	.85	.96	.98	.99	1.00	1.00	4322	2.44

The results of the analysis are displayed in Table 4. It may be noted that since the composition of the restscore differs for each item, the number of observations per restscore group also differs from item to item. The number of observations per restscore group was relatively small for the lower-score groups, and a restscore equal to 0 was only observed for item 8. Thus, most of the information that was relevant for the assessment of monotonicity was obtained from the middle-score to higher-score groups.

For the comparison of manifest monotonicity with its complement, the values of $BF_\mathrm{{MM,NM}}$ ranged from 0.001 to 90,189. Items 1 and 8 had a Bayes factor lower than $\frac{1}{20}$ while all the other items had a Bayes factor higher than 20. Items showing a larger and more stable increase of the proportion of correct responses across the restscore resulted in higher estimates of the Bayes factor. For 8 out of 11 items, the Bayes factor exceeded 1000.

Items 1 and 8 both display nonmonotone orderings. Because the items have multiple choice format, a possible explanation for nonmonotonicity is that particular distractors fail to function for low-ability candidates, resulting in a local decrease of the conditional probabilities. To test the possibility of a floor effect ($H_\mathrm{{F}'}$), we considered the hypothesis that manifest monotonicity only holds for the highest half of the score groups ($\pi _{5}$ through $\pi _{10}$), allowing for possible nonmonotonicities in the lower score groups ($\pi _{0}$ through $\pi _{4}$). Contrasting $H_\mathrm{{F}'}$ with $H_\mathrm{{MM}}$ for each of the 11 items resulted in Bayes factors that showed strong support for $H_\mathrm{{F}'}$ ($BF_\mathrm{{MM,F}'} < 0.0001$) for the two problematic items, while the Bayes factors for the other nine items showed support for manifest monotonicity. For items 1 and 8, the Bayes factor contrasting $H_\mathrm{{F}}$ with its complement showed support for $H_\mathrm{{F}}$, which suggests that the two items may suffer from malfunctioning distractors for low ability candidates.

Because nonmonotone items may confound the restscore, it is advisable to sequentially remove items until no item shows a violation, rather than removing all items with $BF_\mathrm{{MM,NM}} \le \frac{1}{20}$ at once. First, item 1 was eliminated from the test and the procedure was applied again to the remaining items. For item 8, the estimated Bayes factor equalled 0.016, and for the other items $BF_\mathrm{{MM,NM}} \ge 20$. After item 8 was also removed from the test, for eight out of the remaining nine items, $BF_\mathrm{{MM,NM}} \ge 20$, indicating strong support for manifest monotonicity over its complement. However, for item 10, the estimated Bayes factor was equal to 7.11, indicating only modest support for manifest monotonicity. Because item 10 showed strong support for monotonicity in the previous two analyses, we decided to keep this item in the test.

While the values of $BF_\mathrm{{MM,NM}}$ suggest general support for latent monotonicity for the remaining items, one would like to exclude the possibility that there are small local violations of latent monotonicity for these items. For this purpose, the Bayes factor contrasting manifest monotonicity with essential monotonicity was used. Table 4 shows the estimates of $BF_\mathrm{{MM,EM}'}$ for the original set of 11 items. Only three items show support for manifest monotonicity compared to essential monotonicity ($BF_\mathrm{{MM,EM}'} \ge 3$). After the nonmonotone items 1 and 8 were removed the results improved, with seven out of the remaining nine items showing support for manifest monotonicity. The Bayes factors of item 2 and item 10 did not show support for manifest monotonicity compared to essential monotonicity. Thus, the quality of these items and the extent to which they contribute to the reliability and validity of the test should be critically examined. However, the simulation results suggested that this absence of support may also have resulted from lack of power, because support for $H_\mathrm{{MM}}$ relative to $H_\mathrm{{EM'}}$ was not always found for well-functioning items under conditions similar to the current condition ($n = 500, 1000$; $k = 10$). Overall, these results support latent monotonicity for these nine items.

## Discussion

This article proposed a methodology for evaluating the amount of support the data provide in favor of manifest monotonicity, which is quantified using the Bayes factor. The procedure remains neutral with respect to whether the aim is verification or falsification. By determining the support for manifest monotonicity compared to its complement, the procedure provides a general measure of the amount of support for this property. Since the complement of manifest monotonicity is unspecific, the procedure can be supplemented by subsequently comparing manifest monotonicity with an informative alternative hypothesis. Informative alternatives can either serve as alternatives that are of substantive interest (such as the floor effect in the empirical example), or as a way of more extensively investigating the amount of support in favor of manifest monotonicity (such as essential monotonicity in the empirical example). Because the Bayes factor can be determined for any set of order constraints on the conditional item probabilities, the approach is flexible with respect to the range of hypotheses that can be compared.

The simulation results showed that contrasting manifest monotonicity with its complement effectively identified the nonmonotone item. Including a second step in the procedure where manifest monotonicity was contrasted with essential monotonicity helped to identify weakly discriminating items, but mainly for short tests. Longer tests seemed to require larger sample sizes before $H_\mathrm{{MM}}$ and $H_\mathrm{{EM}'}$ can be distinguished sufficiently. This could be an indication that for long tests, it is useful to employ a more liberal version of essential monotonicity—allowing for nonmonotonicities between score groups more than one step removed—in order to successfully differentiate between a completely monotone ordering and approximately monotone orderings of the conditional item probabilities. In addition, these results illustrate that longer tests require larger sample sizes before one can expect to find support for manifest monotonicity relative to essential monotonicity, due to data sparsity in score groups. Thus, for long tests and small sample sizes, removing items that do not show support for manifest monotonicity over essential monotonicity may result in an overly large proportion of well-functioning items being discarded and thus is not advisable. In addition, further research may show that for some applications, having items that are at least essentially monotone might be sufficient. In this case, one could consider contrasting $H_\mathrm{{EM}}$ with its complement, to determine whether there is support for essential monotonicity (rather than manifest monotonicity).

The procedure could be extended to assess monotonicity for a set of items at once. However, this approach runs the risk of masking violations for a particular item if the other items are monotone, so it seems that any global analysis should be followed by an analysis at the item level even if the global analysis indicates overall support for latent monotonicity. Multiple testing does not appear to be problematic, because the simulation study has shown that regardless of test length and sample size the probability of rejecting monotonicity for an item that is monotone appears to be close to 0. Likewise, the probability of finding strong support in favor of monotonicity when a nonmonotone item is evaluated appeared to be close to 0, also suggesting that multiple testing may not be problematic for the proposed procedure, especially if it is used in an exploratory rather than a confirmatory setting.

The Bayes factor provides a measure of *relative* support (Kass & Raftery, 1995), and does not directly inform the researcher about the probability that manifest monotonicity is true but rather about the extent to which this has become more likely after having observed the data. Hence, the Bayes factor provides researchers with an objective assessment of the degree of support in favor or against the hypotheses, which they can use to determine whether they consider a hypothesis to be plausible after having observed the data.

The proposed procedure makes use of an uninformative prior distribution that does not favor any particular ordering of the conditional item probabilities. Because test items are artifacts constructed with the specific purpose of monotonically measuring a specific trait, one could argue that the prior distribution should take this substantive information into account and should to some degree favor monotonic and essentially monotonic orderings over orderings that show large deviations from monotonicity. Such a prior distribution would concentrate its density around the area corresponding to manifest monotonicity. However, such an informative prior would a priori favor the property that is evaluated by the procedure, and this would affect the Bayes factor. We posit that for the assessment of latent monotonicity, a measure of support should solely reflect the extent to which the data (and not the researcher’s prior expectations) support the model assumption, and hence that the use of an uninformative prior should be preferred. We contend that this is consistent with the idea that model assumptions should be critically evaluated and that concerns raised about this assumption should be eliminated not by indicating that items were meant to behave monotonically by the person who designed them, but rather by determining the extent to which the data support this claim. This is precisely what the proposed procedure aims to do.
